# Printing by and for the Blind

**Published:** 1913-03-01

**Authors:** A. W. G. Ranger, E. B. B. Towse, H. M. Taylor, W. P. Merrick

**Affiliations:** Chairman of the Council; The British and Foreign Blind Association, 206 Great Portland Street, London, W.; Captain, V.C., late Gordon Highlanders, Vice-Chairman; The British and Foreign Blind Association, 206 Great Portland Street, London, W.; Chairman of the Technical and Book Committee; The British and Foreign Blind Association, 206 Great Portland Street, London, W.; The British and Foreign Blind Association, 206 Great Portland Street, London, W.


*To the Editor of* The Hospital.

### Printing by and for the Blind.

*To the Editor of* The Hospital.

Sir,—Will you permit us through the medium of your columns to make an appeal on behalf of the British and Foreign Blind Association? We have all been for several years blind members of its Executive Council. We feel, therefore, that we can speak with some confidence both of the desires and requirements of the blind in respect of books printed in embossed type, and also of the capability of the Association to meet the demands at present made on its resources. One of the chief objects of the Association, if not *the* chief object, is the maintenance of a printing press for works in embossed type. The press as at present carried on is, we may say without fear of contradiction, by far the most important of its kind not only in the United Kingdom, but in the Empire. The Council make a practice of giving employment to the blind as much as possible, and a large proportion of the work done on the premises, including stereotyping, printing and binding, is performed by blind people. Constant demands are being made to undertake additional work with which it is impossible to comply.

The Financial Position.

The Council have for a long time felt that their present premises (206 Great Portland Street, W.) are quite inadequate for the work, and they have acquired a more commodious site only a few doors away in the same street. Although the improvements introduced in the printing of embossed books by the Association during the last ten years have called forth expressions of unbounded admiration from the blind, the Council feel that they cannot long continue to carry on their work in a satisfactory manner in their present cramped premises. Some few years ago an anonymous donor, through the hands of the then Chairman of the Council, gave £10,000, of which £9,000 was allocated to the building fund. The fund has since increased by £4,354. The sum of £10,000 has been expended in carrying out part of the work entailed by the scheme for a new building, and the completion of the work, including adequate equipment of the building, necessitates the raising of a further sum of £29,000. The Council are most anxious that the invested funds of the Association, producing an annual income of some £400, should not be touched. To maintain the work on an enlarged scale an increase of £1,000 in annual subscriptions is also urgently needed. It may be mentioned that the

Association is incorporated as a non-profit-making institution, and that no gain is made by the sale of the books and magazines which it publishes. The Association is wholly dependent on voluntary contributions; we confidently recommend it to the kind consideration of the charitable public as an institution well worthy of their support. Donations or annual subscriptions should be sent to the Honorary Treasurer, Mr. Douglas A. Howden, or to the Secretary-General at 206 Great Portland Street, W. It would be a source of great comfort to the Council to be able to announce at the opening of the new building that it was free from debt. Two members of the Council have each promised to give £25 to the general fund for each £1,000 raised for the building fund before July next.

Their Majesties have for some time been patrons of the Association, and the King has lately shown his interest in its work by graciously permitting the announcement to be made that his Majesty hopes to be able to open the new building on its completion.—Yours faithfully,

A. W. G. Ranger, M.A., D.C.L., Chairman of the Council; E. B. B. Towse, Captain, V.C., late Gordon Highlanders, Vice-Chairman; H. M. Taylor, J.P., M.A., F.R.S., Chairman of the Technical and Book Committee; W. P. Merrick.

The British and Foreign Blind Association,
205 Great Portland Street, London, W.,
February 24, 1913.

